# TAPDANCE: An automated tool to identify and annotate transposon insertion CISs and associations between CISs from next generation sequence data

**DOI:** 10.1186/1471-2105-13-154

**Published:** 2012-06-29

**Authors:** Aaron L Sarver, Jesse Erdman, Tim Starr, David A Largaespada, Kevin A T Silverstein

**Affiliations:** 1 Biostatistics and Bioinformatics Masonic Cancer Center, University of Minnesota, Minneapolis, USA; 2 Obstetrics, Gynecology & Women's Health and Masonic Cancer Center, University of Minnesota, Minneapolis, USA; 3 Department of Genetics, Cell Biology and Development and Pediatrics Masonic Cancer Center University of Minnesota, Minneapolis, USA

## Abstract

**Background:**

Next generation sequencing approaches applied to the analyses of transposon insertion junction fragments generated in high throughput forward genetic screens has created the need for clear informatics and statistical approaches to deal with the massive amount of data currently being generated. Previous approaches utilized to 1) map junction fragments within the genome and 2) identify Common Insertion Sites (CISs) within the genome are not practical due to the volume of data generated by current sequencing technologies. Previous approaches applied to this problem also required significant manual annotation.

**Results:**

We describe Transposon Annotation Poisson Distribution Association Network Connectivity Environment (TAPDANCE) software, which automates the identification of CISs within transposon junction fragment insertion data. Starting with barcoded sequence data, the software identifies and trims sequences and maps putative genomic sequence to a reference genome using the bowtie short read mapper. Poisson distribution statistics are then applied to assess and rank genomic regions showing significant enrichment for transposon insertion. Novel methods of counting insertions are used to ensure that the results presented have the expected characteristics of informative CISs. A persistent mySQL database is generated and utilized to keep track of sequences, mappings and common insertion sites. Additionally, associations between phenotypes and CISs are also identified using Fisher’s exact test with multiple testing correction. In a case study using previously published data we show that the TAPDANCE software identifies CISs as previously described, prioritizes them based on p-value, allows holistic visualization of the data within genome browser software and identifies relationships present in the structure of the data.

**Conclusions:**

The TAPDANCE process is fully automated, performs similarly to previous labor intensive approaches, provides consistent results at a wide range of sequence sampling depth, has the capability of handling extremely large datasets, enables meaningful comparison across datasets and enables large scale meta-analyses of junction fragment data. The TAPDANCE software will greatly enhance our ability to analyze these datasets in order to increase our understanding of the genetic basis of cancers.

## Background

Forward genetic screens have opened doors to completely novel areas of biological understanding [1]. Starting with fundamental work with yeast, a significant portion of our molecular understanding is built on hypotheses originating from unbiased screens. Recently, forward genetic screens have been used to study the genetics of cancer in vivo, first using murine retroviruses [2] and more recently using mobile genetic elements or transposons engineered to be active within the murine genome [3]. Transposons are mobilized by the presence of a transposase enzyme, which can be expressed in a tissue specific manner leading to the formation of tissue specific cancers [4,5]. In the simplest possible conceptual model, tumors may be formed by the disruption of a tumor suppressor gene or activation of an oncogene by a transposon, which then leads to a selective growth advantage for the cell and progeny cells that harbor the insertion. Identification of Common Insertion Sites (CISs) in a significant number of independent tumors strongly implicates genomic regions to be fundamentally involved in tumor formation. Prior work has described methods to identify CISs as well as associations between CISs in datasets [4-9].

Identification of the location of transposon insertion requires obtaining the sequences crossing the junction between the transposon and the authentic genome sequence [10]. Mapping the genomic sequence adjacent to the transposon to a reference genome allows the determination of the specific site of integration. Large numbers of insertion sites can be identified in tumors. Next generation sequencing technology has allowed massive numbers of transposon-genomic junction fragment to be sequenced. The sheer quantity of sequences has introduced unanticipated problems to pipelines designed to identify CISs.

The goal of the TAPDANCE software is to fully automate the analysis of statistically significant CISs using next generation sequencing of insertions from samples where a mobile genetic element has been activated within the genome. In murine forward genetic cancer screens, these CISs represent potential drivers of tumorigenesis. The effort and expense required to validate potential oncogenes and tumor suppressors in human cancer is immense. Yet, the potential benefit to the treatment of these diseases and reducing human suffering cannot be understated. Thus, it is of paramount importance to identify and rank the importance of the driver regions (CISs) of the genome in these datasets. To this end we have developed the software described here, and made it publicly available.

## Implementation

**Figure 1:**
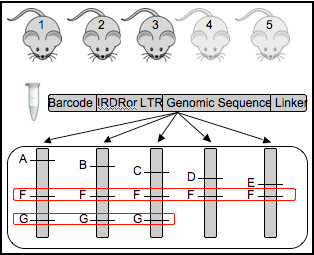
**Visual summary of the TAPDANCE process.** Libraries of inserts from sets of mouse tumors are generated and sequenced. Barcode, IRDR and linker sequences are trimmed, and the remaining genomic sequence is mapped to the genome. Regions of insertions overrepresented within the genome and the statistical probablility of observing such events are determined in an automated manner allowing the comparison and contrasting of multiple datasets. Genomic loci may be common among many mice (e.g. F) or just a subset with a common observed phenotype or inherent genotype (e.g., G).

**Figure 2:**
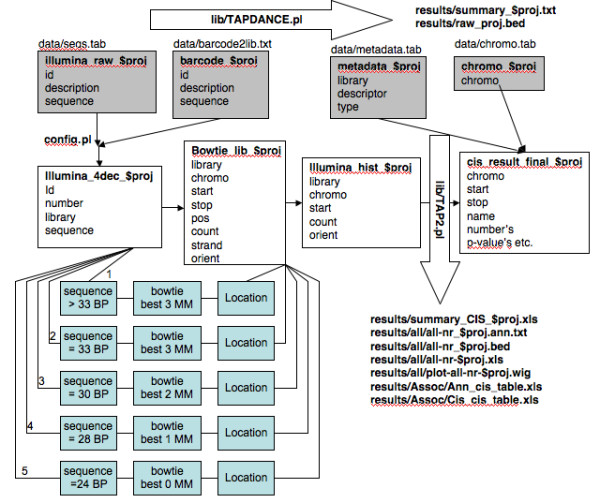
**TAPDANCE database schema and processing flowchart.** Overview of the TAPDANCE process. Input files are loaded into the database tables named with the project id. SQL and perl functions are used to identify library of origin, genomic sequence, remove duplicate sequences and to allow insert location identification using the bowtie mapping algorithm. This mapping process is iterative in the first iteration sequences > 33 bp are mapped allowing 3 mismatches. Anything that did not map in the first round was remapped following removal of the 3’UTR to leave only 33 bases in the second round. Similarly in the 3^rd^ round remaining unmapped sequences of 30 bp were mapped allowing 2 mismatches. In the 4 th round previously unmapped sequences of length 28 bp were mapped with 1 mismatch. Finally previously unmapped sequences of length 24 bp are mapped with 0 mismatches. The mapped data is summarized and finally exported by the TAPDANCE.pl script using configurable data stored in the config.pl script including barcodes and insertion derived sequences. The TAP2.pl scripts assembles sets of inserts, conducts CIS analyses, Co-CIS and Pheno-CIS analyses resulting in exportable files containing relevant information. All file locations are shown relative to root and additional intermediate tables are generated during processing as documented within the various scripts and dependencies. Persistent tables and results files are named using the $proj variable which is set in the config.pl file.

The processing and analysis pipeline takes as input 4 files (examples included in Additional file 1: Table S7 Full examples in Additional file 2). The first file is the sequence file produced by the high-throughput sequencing machine. Each line in this file contains a unique sequence identifier and the sequence read. An informative sequence read will consist of a DNA barcode sample-identification sequence ligated to either the Inverted Repeat Direct Repeat (IRDR) for SB studies or Long Terminal Repeat [LTR] for proviral studies, followed by the captured genomic sequence and potentially ending with the ligation adapter/linker sequence (see Figure 1). The second file contains the barcode sequences mapped to library names. In this paper insertion libraries are generated from tumors thus the word tumor and library is used interchangeably throughout. The third file contains a set of metadata characteristics describing the sets of libraries for CISs analyses (e.g., tumor types, genotypes or phenotypes). The last file contains a list of chromosomes to potentially exclude from the analysis. Using these four input files the software automatically performs raw sequence processing and trimming, mapping to the reference genome, CIS identification, and nearest-gene annotation as described below. A general overview of the process including a partial database schema is provided as Figure 2.

### Raw sequence processing and trimming

The generation of PCR products containing the junction fragments between transposon insertion sites and the flanking genomic sequence has been described in detail previously [4-6]. The resulting PCR products are then sequenced utilizing next generation sequencing to generate hundreds of thousands (454 sequencing) or millions (illumina sequencing) of reads 75-100 bp in length. These sequences are loaded at the outset into a relational database and database queries (SQL) are used to identify and remove barcodes denoting the library of origin. Additionally, transposon sequences and linker fragments are trimmed off, leaving only endogenous genomic sequence. In order to speed up the mapping process, identical sequences that are derived from the same library are condensed to a single sequence entry, retaining the total count of observations made.

### Sequence mapping to the reference genome

**Figure 3:**
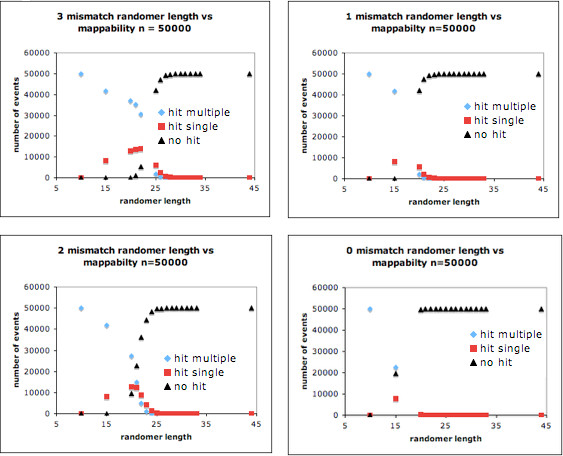
**Genomic mapping and sequence length.** Randomly generated sets of 50,000 (A|G|C|T) sequences of varying lengths described on the X-axis were mapped to the mouse genome using bowtie. The total number of mappings of each sequence that either mapped to no region (black triangle) mapped to multiple regions (blue diamond) or mapped to a single region (red square) were plotted for each sequence length using bowtie mapping software allowing A) 3mismatches B) 2 mismatches C) 1 mismatch and D) 0 mismatches. At intermediate sequence lengths for a fixed number of mismatches, ~20 % of randomly generated sequences were capable of uniquely mapping to the genome.

Genomic sequences are then mapped using the bowtie algorithm [11]. To ensure that mapping to the genome is due to authentic transposon insertion, we required that mapping commence directly 3’ to the transposon sequence. For SB transposon mapping, the flanking genomic sequence is required to start with a TA dinucleotide. This was done in order to minimize the potential for multi-mer ligation leading to incorrect inference of transposon integration. To minimize false positive mapping, we mapped randomly generated sets of 50,000 DNA sequences using bowtie and determined the minimum sequence length required so that none of the randomly generated sequences mapped to the mouse genome. The empirical cutoffs established by this analysis were 33 nucleotides for 3 mismatches, 30 for 2 mismatches, 28 for 1 mismatch and 24 for 0 mismatches (Figure 3). It was surprising that ~20% of the randomly generated sequences map to the genome uniquely if mapping length mismatch thresholds were relaxed below these cutoffs. To avoid this random mapping behavior and increase the stringency of the analyses, genomic sequences of 23 or fewer bases are discarded.

The importance of considering sequence length and mapping directly to the first base following the transposon increases as the total number of sequences examined increases. Since partial linker sequences and unintended ligation products may remain on the 3’-end of full length junction sequences, we developed an iterative mapping process in order to maximize authentic mappings and minimize the potential for mismapping (Figure 2). In the first iteration, full-length sequences are mapped allowing 3 mismatches if their length is >33. In the second iteration sequences that did not map in the first round of mapping had their 3’ ends removed to leave only the first 33 bases. These 33 bases were then mapped allowing 3 mismatches. In the third iteration, sequences that did not map in the second iteration were shortened to 30 bp and combined with shorter sequences before mapping was again attempted allowing 2 mismatches. The procedure was then repeated using sequences of 28 bp (1 mismatch) and 24 bp (0 mismatches).

Mapped regions are exported to a bed file in order to visualize all the “raw” mappings and the strand on which the mapping was observed using genomic browsers such as IGV [12] or for smaller datasets, the UCSC genome browser [13].

### Mapping orientation

**Figure 4:**
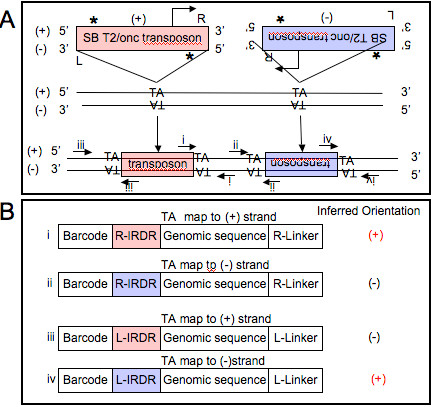
**SB transposon mapping and Orientation.A**) The SB transposon is asymmetric and can map in either + or – orientation within specific TA sites. **B**) Genomic region after insertion of SB transposon in either + orientation (red) capable of driving transcription of genes encoded on the positive strand or in – orientation (blue) capable of driving genes encoded on the – strand. In either direction a splice site acceptor is available, leading to transcript disruption. PCR products derived from SB junction fragments are capable of determining which orientation the transposon has integrated. The sets of primers i-iv indicate how the PCR products are obtained. Each of the transposon side primers contains a library specific barcode to allow multiplexing during the sequencing process.

The SB transposon and MULV retrovirus are both asymmetric, meaning that the biological effect of the insertion depends on the orientation of the insertion. For this reason it is important to track the orientation of the insertion. Generally, when insertion-genomic junctions are amplified, two separate PCR reactions are performed, one that amplifies junctions from the “right” side of the insertion and a separate reaction that amplifies junctions from the “left” side of the insertion. This procedure is used to maximize the chance of recovering sequence from an insertion if the required restriction enzyme site is not present on either side of the insertion. The sequences from these two PCR reactions are prepared separately for the right reads and the left reads. This is relevant because the SB transposon can activate transcription only if pointed in the correct orientation, whereas a splice site acceptor capable of causing transcript disruption is present in both orientations (Figure 4A). Furthermore, genomic sequence derived from both right and left library generation can map to the + or – strand. Specific insertion orientation to the positive strand can be identified by either a right read that maps to the + strand or a left read that maps to the – strand. (Figure 4B) Alternatively, specific transposon insertion orientation to the negative strand can be identified by the opposite events. To simplify the analyses, all insertions are converted so that the site of integration is defined on the strand that can drive transcriptional activation. In other words if the SB transposon is mapped to the + strand it can drive transcription on the positive strand and if it is mapped to the negative strand it can drive transcription on the negative strand.

### Elimination of non-clonal insertions

Several artifacts are present in the sequence datasets generated by PCR amplification of insertions. The first artifact is due to remobilization of a clonal transposon insertion into a neighboring TA dinucleotide in a small subset of daughter cells of the original clone. This artifact can be attributed to “local hopping” of the remobilized transposon [14]. In the dataset this artifact presents as a dominant TA with many mapped reads and several neighbouring TA’s with relatively few mapped reads. We hypothesize that these insertions were originally in the dominant TA, and then jumped to the neighbouring TA in a daughter cell. Therefore, these insertions should be grouped with the dominant TA. Another artifact is created when sequencing errors at the beginning of the mapping sequence leads to incorrect mapping to TA positions only a few bases away. This artifact also presents as a dominant TA with a few neighboring TA’s being mapped at a very low frequency. To minimize the impact of counting these non-clonal events as unique events, the raw data counts are binned into 100 bp regions based on the location and orientation of insertion.

Finally, transposon activity is not extinguished as the tumor develops in a forward genetic screen. This means that new insertions will continually occur in daughter cells of the original clone that forms the tumor. Insertions that occur later in the development of a tumor will only occur in a few cells and should be considered passenger mutations. This effect appears in the dataset as mapped insertions that are only counted once or twice, as opposed to clonal insertions that are counted many times by the NGS machine. In order to exclude these low-level passenger insertions, we set a cut-off based on the percentage of the total mappable reads in each tumor. For example, consider a case where 100,000 sequence reads from a single tumor can be uniquely mapped to 1,000 regions in that tumor. In our datasets we find that a subset of the 1,000 regions will be identified by a single sequence read while the remaining regions will be identified by many sequence reads. To set a cut-off we require that a region be identified by, at least, a certain percentage of the total sequence reads that were mapped to that tumor. If we set the cut-off to 1/10,000^th^ that would mean that only the subset of 1,000 regions that were identified by at least 10 reads will be included in CIS analysis. This cut-off eliminates mapped insertions that are only read a few times and probably represent non-clonal insertions. TAPDANCE allows the user to select the cut-off value. Alternatively, the user can choose not to have a cut-off. Based on our analysis of datasets that directly compare the 454 platform with the Illumina GAIIx platform we recommend using no cut-off for 454 data and a cut-off of 1/10,000^th^ for Illumina data.

### Project summary files

A summary file is generated for each project that describes the counts for each project, for each directional library, and for each library. The percentage of mappable reads that map to the genome for each run and broken down by library can be obtained from this file. All sequences from a single sequence run are deposited into the SQL database.

### Meta-analyses of multiple projects

Each project can be merged with any number of previously existing projects in the database, and files containing all raw mappings and all regional mappings at the thresholds defined in the config.pl file are automatically generated. The software will merge libraries that are identified by the same name, and multiple projects can utilize the same sets of barcodes for different projects without causing confusion, since the informatics model utilizes the library name as a unique identifier.

One of the major bottlenecks in our previous workflows was the combination and reanalysis of datasets that were obtained in separate sets of sequencing runs. To simplify this informatics problem we wrote the software to allow combination of any set of projects to generate a new project or meta-analyses to allow CIS generation from any subset of libraries. In this manner a common set of barcodes can be reused over and over in separate projects and the individual projects can be efficiently analyzed together.

### CIS calculation

The identification of CISs based on large numbers of sequences from hundreds or thousands of libraries is a nontrivial statistical and informatics challenge. TAPDANCE uses Poisson distribution statistics to calculate CISs. The Poisson distribution determines the probability of observing a given number of events within a region assuming that these events occur in an essentially unbiased manner. In this case the transposon insertions are the events, while the expected number of insertions in a given window is calculated based on the size of the genome and the total number of insertions. The probability estimate is then corrected based on the total number of windows examined (i.e., the total number of insertions) using Bonferroni correction to account for multiple testing. Essentially, the probability of observing a given number of insertions becomes smaller as the genome becomes larger, or the window of interest becomes smaller, or the total number of insertions becomes smaller, or the number of insertions within a given window becomes larger.

More formally the Poisson distribution is calculated using the following formula.

(1) $$\text{P}\left(\text{x}:\text{u}\right)=\left({\text{e}}^{-\text{u}}{\text{u}}^{\text{x}}\right)/\text{x}!$$

Where

e, is the base of the natural logarithm; x, is the actual number of inserts within a window; u, is the number of inserts observed (total) / (genome size /window size).

Each dataset is analyzed multiple times using different window sizes, which generates a list of CISs for each window size analyzed. For each CIS analysis, the window sizes to be used are determined by identifying the maximum window size that will give a corrected p-value < 0.05 (after correcting for multiple testing) with an integer number of insertions by examining window sizes from 10000 bases to 301000 bases in 1000 base steps. If fewer than 2000 insertions or more than 200000 inserts are examined, default window sizes of 12500, 25000,50000,100000, 200000, and 301000 bases are used.

**Figure 5:**
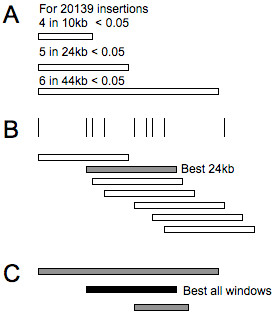
**Window sizes, CIS calculation and resolution of overlapping CISs.A**) Based on the total number of insertions being analysed for CISs, Window sizes (10,000-301,000 bp) are calculated to define the largest window size which is capable of showing a significant CIS (Poisson distribution p-value < 0.05 following bonferroni correction based on total window size) using total insertion numbers which can only exist as integer values. Only the first 3 window sizes for 20139 insertions are shown here. **B**) For each of the window sizes the p-value is calculated for each possible window based on the total number of insertions starting with every insertion throughout the genome. Non-overlapping windows with the lowest p-value (most insertions) are then chosen for each window size where the p-value is below a user-defined threshold. **C**) In order to combine the different window sizes, non-overlapping windows with the lowest p-value are chosen and these are returned as CISs. In the case shown, the 24 kb window with 7 insertions had a lower p-values than best 44 kb window and the best 10 kb window within the region.

A peak finding algorithm was written to iteratively identify non-overlapping peak windows with the largest number of events for each window size. The dataset is then analyzed using each different window size, which generates a list of CISs for each window size analyzed. Since the use of multiple window sizes can lead to multiple potential overlapping CIS definitions, these are resolved by identifying the minimal set of non-overlapping regions that have the most significant probability. If the p-values for two regions are identical, the larger window is used as the default (Figure 5).

### Three methods to count inserts to determine p-value

**Figure 6:**
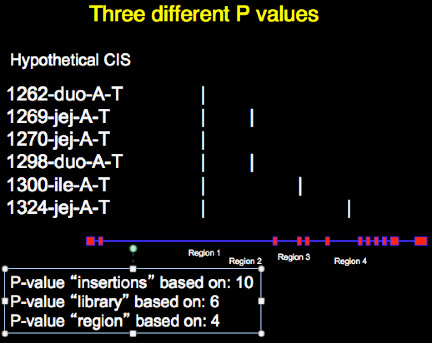
**Insert counting methods for p-value calculation.** Three different methods of counting the number of insertions within a given CIS are used by the software in order to remove potential artifacts from the final CIS list. In the figure 10 transposon insertions from 6 different tumor libraries are shown. The number of insertions can be derived 1) from the total number of inserts 2) the total number of libraries within the CIS and 3) the total number of unique regions within a cis that hold an insertion. The total number of inserts obtained by these 3 counting methods are then indivually used to test the null hypothesys that no enrichment is present using the Poisson distribution based on the window size, the genome size and the total number of inserts present in the dataset being examined. We expect Bonferroni corrected p-values to be less than 0.05 for each of these 3 methods of counting in order to define ideal CIS.

The total number of events (insertions) is required in order to use the Poisson distribution to calculate a P-value. We propose to use three different methods to determine the number of events in a transposon insertion dataset (Figure 6). These three methods test 3 different null hypothesys. The first method uses the total number of unique mapped insertions as the event count. We refer to this method as “insertion p-value” and test the null hypothesys that the total number of inserts within a region (independent of library of origin or insert location) is similar to what could be expected to be observed by random chance. In the example CIS depicted in Figure 5 the total number of events used to calculate the p-value is 10. The potential problem with this approach is that a single library can have multiple insertions in a given genomic window. As discussed above, this could be caused by transposon remobilization or local hopping. Counting these local hopping insertions as separate unique insertions would be misleading. To overcome this effect, we use a second method that uses the total number of unique libraries within a region, referred to as “library p-value” and test the null hypothesys that the total number of libraries within a region (independent of total number of inserts or insert location) is similar to what could be expected to be observed by random chance. This method counts multiple insertions from the same tumor as a single insertion if they occur in a single genomic CISwindow. In Figure 5 the total library count is 6. With this method another misleading situation can arise when many libraries map to the same 100 bp region, Using the example in Figure 5, sequencing data indicates that six tumors have a mapped insertion in a single TA in region 1. Unless there was strong selection for this single TA, which we hypothesize is generally not the case, this represents an artifact. This artifact could arise from false priming events or barcode mis-assignments. In order to eliminate these possible artifacts, we also use a third method to calculate the p-value, referred to as the “region p-value” and test the null hypothesys that the total number of regions (independent of library of origin or total insert count) is similar to what could be expected to be observed by random chance. This method uses the total number of unique regions within a genomic window as the total number of events. “Strong” CISs, meaning CISs that are composed of many insertions, libraries and regions will be statistically significant using all three methods described above. “Weaker” CISs, however will be significant only using one or two of the methods. We recommend that at a minimum, a CIS should have a insert p-value, a region p-value and a library p-value less than 0.05 following Bonferroni correction. The p-value test for each of these 3 events is user definable, with a default setting of 0.05.

### Donor chromosome considerations

The user is provided with the option of excluding chromosomes from consideration due to the potential for local hopping observed within the donor chromosome (the chromosome where the transposon resides prior to mobilization).

### Annotation

CISs are then automatically annotated based on a user supplied .bed file to provide the names of the genes surrounding the regions of interest. All elements within 20,000 bases are returned to the gene name column. If no elements are present, this field gets filled with the default statement “No results within a 20,000 bp window”.

Every project for which this software has been utilized has required analyses of multiple overlapping and non-overlapping sets of libraries. To facilitate these analyses, the software generates CISs based on a model where the library and the analysis group are defined. Any library within a project can be combined with any other libraries, resulting in the automatic calculation of new combined wig files of raw mapping regions, insertion regions and CISs.

### Co-CIS and Pheno-CIS analyses

Valuable biological information can be extracted from the CIS datasets by determining the association between phenotypes and CISs (Pheno-CISs) as well as the associations between individual Cooccurring CISs (Co-CISs). TAPDANCE will automatically generate the associations between the different phenotypes, between phenotypes and CISs, and between separate CISs using Fisher’s Exact test. Fisher’s exact test compares the probability of getting the observed data distribution and all more extreme deviations relative to the null hypothesis, where the proportions between the two groups are random. Multiple testing correction procedures with varying levels of rigor (Bonferroni and Benjamini Hochberg) are then applied separately to each of the three types of associations being tested based on the number of tests conducted. For the determination of association between phenotypes and CISs the total number of tests being calculated can be computed by multiplying the number of phenotypes tested by the number of CISs being tested. For the determination of association between CISs (or between phenotypes) the total number of associations tested can be computed using the formula to calculate the total number of edges and diagonals in a polygon of n sides (total = n(n-1)/2) where n is equal to the number of CISs or the number of phenotypes. It is important to emphasize that examination of association in datasets or parts of datasets with insufficient power may mask significant events due to the necessity to correct for multiple testing. The number of CISs to test for association can be set by the user by defining a maximum p-value for CISs to be considered in association analyses. It is suggested that the user be aware of the power available based on the sample size with regards to the annotations and CISs examined. The default setting is all CISs with a p = value < 10e-5. The phenotypes used for this calculation are all the sets where CIS generation is calculated within a given project as defined by the user.

## Results

A number of tests were used to determine that our automated package was performing robustly. Genomic positions were randomly generated and assigned to libraries, with library counts equal to those from real experimental data sets. Analyses of CISs in these random datasets determined that no genomic windows were statistically significant. We further tested authentic mapped mouse sequences obtained from chip-seq input control experiments with library and sequence counts equal to those from real experimental data sets. Somewhat surprisingly, analyses of CISs in these datasets identified several regions with high significance, which were repeatedly found to be present. As these datasets are defined by examining sequences in the absence of any selective pressure, we hypothesize that these are regions that are present in high copy number within the murine genome but they are mistakenly annotated as a single copy in the genome build. This hypothesis is supported by other groups [15]. As these regions are also observed in many of the screens we have conducted, we believe that these regions should not be considered as drivers of tumorigenesis. We also found another artifact specific to transposon screens that contain endogenous murine sequence within the transposon. Due to local hopping within the original donor concatamer, the LM-PCR protocol will amplify these internal jumps and they will map to the murine genome. For example, part of the EN2 murine gene sequence is included in the T2/Onc transposon. In SB screens using this transposon, insertions mapping to EN2 are excluded because of this artifact. All known artifact regions, such as these, are labeled with the string 'BAD' in the annotation bed file and are not reported by the software. The excluded regions are included as Additional file 1: Table S1.

To compare the full functionality of TAPDANCE with previous methods we reanalyzed the sequences obtained for previously published colon cancer and liver cancer screens [3,4]. Running the process using TAPDANCE takes a fraction of the time of the original process. These datasets were generated using the Roche 454 GS/Flex platform. A representative summary of the 4 required input files is provided as Additional file 1: Table S7. Summary statistics are presented for the dataset as a whole (Additional file 1: Table S8A) as well as broken down by barcode (Additional file 1: Table S8B) and finally by library (Additional file 1: Table S8C).

The CIS thresholds automatically calculated via iterative poisson distribution statistical analyses are very similar to those defined by Monte Carlos simulation and are calculated almost instantaneously (Additional file 1: Table S2).

The CIS list is generated as a spreadsheet that contains the chromosome, start and end address of the CIS region, the 3 associated p-values (insert, library, and region), the libraries that are included, and the annotation. Additionally the total count of inserts that are capable of driving insertions on the positive strand are provided for each CIS. This number can be combined with the total number of insertions to determine whether a specific gene is potentially being overexpressed or is being disrupted due to the orientation of transposon insertion.

**Figure 7:**
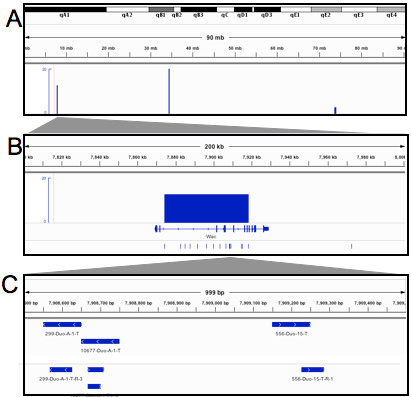
**Analyses of colon cancer data set.A**) CISs on chromosome 18, identified by the TAPDANCE system visualized by IGV , The x-axis shows the position on chromosome 18 and the y-axis shows the –log of the p-value. **B**) Zoomed in visualization of the Wac CIS region on chromosome 18. The CIS region, the intron exon boundaries of WAC and the actual transposon insertion regions are shown. **C**) A further zoomed-in 1000 bp region of the WAC CIS showing further detail regarding the transposon insertion orientation as well as the raw read mappings that were used for the CIS region calls.

CIS regions were identified in the colon cancer data (n = 67; Additional file 1: Table S3) as well as the liver cancer data (n = 27; Additional file 1: Table S4). In total, three files are useful for CIS visualization: (i) the wig file containing the CISs where the CIS regions are identified as peaks with a height corresponding to the –log of the p-value. (ii) a bed file containing the regions and orientation of the insertions used for CIS calculations (iii) a bed file containing all individual seqeunce mappings. These can be loaded into IGV (5) and the specific rational for each CIS, the orientation of each transposon and the raw insertion mappings can be directly analyzed (Figure 7).

**Figure 8:**
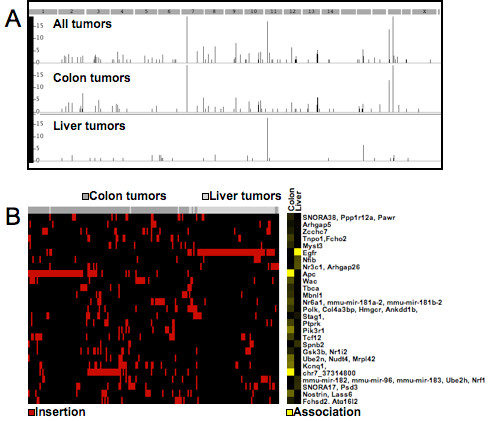
**Genome-wide association of phenotypes and CISs.A**) Genome-wide map of CISs calculated using all tumors, colon tumors or liver tumors. The –log base 10 of the CISs p-value is plotted with an upper threshold of 16. **B**)Heat map of CISs with p-value > 10-5 calculated using all tumors. The header bar indicates colon tumors in dark grey and liver tumors in light grey. Transposon insertions in CIS regions within a given library are indicated by red boxes in the first panel. In the second panel the Fisher’s exact test p-value has been converted to the –log base 10 is plotted as a heatmap with increasing yellow intensity showing increased statistical significance. The CIS containing the Apc gene is highly significantly associated with colon tumors, while a CIS containing the Egfr gene is highly associated with the liver tumors. Actual p-values for association are provided in Additional file 1: Table S6.

To demonstrate the meta-analyses and Pheno-CIS portion of the software we performed a combined analysis of the insertions derived from liver tumors together with the colon cancer tumor insertions and identified CISs (n = 93; Additional file 1: Table S5) in the combined dataset. The TAPDANCE software then automatically calculated associations between tumor phenotypes (colon or liver) and CISs. (Figure 8; Additional file 1: Table S6) Specific CISs showed clear associations with each of the tumor types as would be expected from the known biology. For example insertions in APC are associated with colon tumors, while EGFR insertions are associated with liver tumors. Additionally, prior to the removal of the Y-chromosome artifact from CIS consideration the male phenotype was commonly observed to be significantly associated with Y chromosome inserts, which shows that the methodology is capable of identifying statistical associations of biological relevance.

To further demonstrate the co-CIS capability of the software we analyzed an insert dataset derived from retroviral insertions obtained from the RTCGD [16]. This dataset composed of ~6700 mapped insertions derived from ~1600 tumor libraries was analyzed for CIS using the TAPDANCE methodology. The results are consistent with previously generated results (Additional file 1: Table S9) and co-CIS were calculated between CIS with a region p-value < 0.00001. The top hits obtained by alternative formulations of co-occurring insertion event analyses [7,9] in this dataset were also obtained by TAPDANCE coCIS analyses (Additional file 1: Table S10).

Finally, we compared our CIS calculation method directly with the modified Gaussian kernel convolution framework [6] using a pancreatic cancer dataset recently published [17]. Our method was able to find and score 18 of the top 20 regions within the top 31 CIS (Additional file 1: Table S11). All top 20 CIS were scored within the top 50 CIS by TAPDANCE. The majority of the differences can be attributed to the different methods of ranking used in the final sorts. It should also be noted that the sequence analyses and genome mappings were done independently, indicating that the full TAPDANCE pipeline is showing similar results to the modified Gaussian kernel convolution method.

## Discussion

Previously, multiple steps in CIS generation sequence cleaning, mapping to the genome, CIS calling, and association needed to be carried out separately in a non-automated fashion. In this work we present a fully automated rationally derived method for complete analyses and make it publicly available. Previously, Monte Carlo simulation based on the actual TA density within the genome as well as the number of inserts on each chromosome was used to estimate the number of inserts necessary to be called a CIS within a given window. Our method, utilizing the Poisson distribution based calculation directly provides similar results (Additional file 1: Table S2) in a far less computationally intense and fully automated fashion. In practice, window size thresholds and significance cutoffs are remarkably similar to Monte Carlo simulation based on TA density on a chromosome by chromosome basis and calculated in a fraction of the time. Additionally, P-values are generated for each CIS, which is not possible using the Monte Carlo method as previously implemented. The incorporation of multiple schemes of counting of inserts for use within the p-value calculation based on 1) total number of inserts within a window; 2) based on the total number of libraries; 3) the total number of unique insert regions allows for the selection of CISs with “ideal” tumor driver characteristics to be ranked higher than CISs with less ideal characteristics.

We show that the potential for incorrect mapping exists with short sequences, and resolve this by requiring input sequences to be of sufficient length such that no sequences will be mapped by random chance. Additionally we have shown that 1) our empirically derived statistical model for CIS calling behaves adequately using random positional/library data and 2) real bias exists in murine sequencing data that needs to be taken into account in the analyses of CISs.

This methodology is robust, statistically conservative and efficient. The results generated are consistent with previous workflows, both in the statistical thresholds identified as well as the CIS list membership. This method has a number of advantages. It runs much faster, and is capable of running the volume of data currently being generated via current NGS techniques. Similar results are obtained, independent of sequencing depth once a basic sampling threshold is reached. Using this system we are able to obtain similar CIS results from datasets that are sequenced separately using 454 (~1000’s of sequences per library) and Illumina (~100,000’s of sequences per library). We have utilized this software in the analyses of ~20 different forward genetic screens using both 454 and Illumina based sequencing approaches.

## Conclusions

Widespread incorporation of this software will allow meaningful meta-analyses of transposon based genetic screens. We also note that the software can be readily configured to identify CISs and associations from any organism.

## Availability

We have made the full software package publicly available at Source Forge (http://sourceforge.net/p/tapdancebio/home) and the Galaxy Toolshed (http://toolshed.g2.bx.psu.edu).

## Requirements

The command line version of TAPDANCE requires the user to have Bowtie [11], PERL DBI and R software installed. The command line version also requires access to a read /write mysql account.

## Competing interests

The authors declare that they have no competing interests.

## Authors’ contributions

ALS wrote software, carried out analyses, and authored paper. JE wrote software, ported software to galaxy and generated documentation at Sourceforge. ALS, TKS, DLS and KATS participated in conceptual discussion, software design, and helped to draft the manuscript. All authors read and approved the final manuscript.

## Supplementary Material

Additional file 1 **Additional supporting Tables.** Table S1.xls. Suspect regions identified in Chip-SEQ data. CISs found to be highly significant in data sets composed from real mouse sequence obtained as a control for CHIP-SEQ randomly assigned to libraries. The trial was repeated with 3 different subsets of data A, B, C. Regions returned from all 3 tests are labeled “BADrepeat” and not returned as CIS drivers. Table S2. Comparison of window sizes and insert numbers calculated to be significant by Poisson distribution followed by Bonferroni correction with window sizes and inserts calculated by Monte Carlo Simulation for Colon cancer dataset and for liver cancer dataset. Table S3. CISs calculated by TAPDANCE method for colon cancer dataset. Table S4. CISs calculated by TAPDANCE method for liver cancer dataset. Table S5. CISs calculated by TAPDANCE method for combined datasets. Table S6. Association results for the combined datasets. Highly significant results are shown in Bold for the association between A)phenoCIS and B)coCIS. Table S7. Examples of 4 files required in the data directory in order to run the command line version of TAPDANCE. A) a file containing sequences labeled seqs.tab. B) A tab delimited file containing the barcodes, the library names ending in either –L or –R based on the direction of priming and the direction of priming (Left or Right). C) A tab delimited text file containing groups for CIS analyses, the default superset for association should be labeled “all” and subsets should be named with 6 or less meaningful characters. D) a Text file containing chromosomes that should not be analyzed due to the presence of the donor transposon concatamer and local hopping. Table S8. Report of the counts of the initial mapping and how many sequences have the described characteristics A) for the entire dataset, B) broken down by directional library, and C) total for each library following combination of left and right primed reads. Additionally in C the number of reads that were mappable, the number of reads that map and the total of regions that map at the defined threshold of the total mapped sequences is reported. For mapping to the genome we have observed 50-80% mapping to the genome of the mutagenized organism. Mapping percentages significantly lower would indicate potential problems. Table S9. CIS identified in the RTCGD retroviral insertion dataset. Table S10. Co-CIS identified in the RTCGD retroviral insertion dataset. Table S11. CIS identified in a pancreatic ductal adenocarcinoma SB screen generated by TAPDANCE methodology directly compared to the TOP 20 CIS generated by the modified Gaussian kernel convolution framework. Click here for file

Additional file 2 **Example data.** We have included a zipped up archive.zip which contains 4 data files in the data directory, as well as the results obtained after running the scripts. Click here for file
